# Identification of Multiple Ingredients for a Traditional Chinese Medicine Preparation (Bu-yang-huan-wu-tang) by Liquid Chromatography Coupled with Tandem Mass Spectrometry

**DOI:** 10.3390/molecules180911281

**Published:** 2013-09-12

**Authors:** Lee-Hsin Shaw, Wei-Ming Chen, Tung-Hu Tsai

**Affiliations:** 1 Institute of Traditional Medicine, National Yang-Ming University, Taipei 112, Taiwan; E-Mail: lychee0130@hotmail.com; 2 Division of Gastroenterology and Hepatology, Department of Internal Medicine, Chang Gung Memorial Hospital, Chiayi 61363, Taiwan; E-Mail: b8902030@gmail.com; 3 Graduate Institute of Clinical Medical Sciences, College of Medicine, Chang Gung University, Taoyuan 33302, Taiwan; 4 Graduate Institute of Acupuncture Science, China Medical University, Taichung 40402, Taiwan; 5 Department of Education and Research, Taipei City Hospital, Taipei 10629, Taiwan

**Keywords:** bu-yang-huan-wu-tang, herbal analysis, LC-MS/MS, traditional Chinese medicine

## Abstract

Bu-yang-huan-wu-tang (BYHWT) is a popular Traditional Chinese Medicine formula consisting of seven herbal medicines (Astragalus membranaceus, Angelica sinensis, Paeonia lactiflora, Ligusticum chuanxiong, Carthamus tinctorius, Amygdalus persica and Pheretima aspergillum), that has been used in China for centuries to overcome stroke-induced disability. To ensure the consistency of quality, a reliable analytical method is required, therefore, we developed a liquid chromatography with tandem mass spectrometry (LC-MS/MS) method for quantitative analysis of the major constituents in BYHWT. The herbal ingredients consisting of the cycloartane-type triterpene glycosides of astragaloside I, astragaloside II and astragaloside IV; isoflavones of formononetin, ononin calycosin, calycosin-7-*O*-β-d-glucoside; ligustilide and paeoniflorin were separated on a C18 column with gradient elution of methanol/10 mM ammonium acetate buffer–formic acid (100:0.1, v/v). This study was performed by a mass spectrometer using electrospray ionization (ESI) with positive ionization ions monitored in the multiple reaction-monitoring (MRM) mode. The linearity, accuracy, precision, limit of detection (LOD) and lower limit of quantification (LLOQ) were validated for this quantification method, and the sensitivity, reliability and reproducibility were all confirmed. The experiments provided a good method for analyzing BYHWT extracts. This study also quantitated the active components in various brands of commercially available products. The results indicated that the pharmaceutical industrial products of BYHWT exhibited considerable variation in their contents of the herbal compounds.

## 1. Introduction

Bu-yang-huan-wu-tang (BYHWT) was originally described in the traditional herbal literature of Yi-Lin-Gai-Guo written by Qing-Ren Wang in 1830 during the Qing Dynasty. The decoction is comprised of seven commonly used Chinese herbal drugs: (1) Radix Astragali (Chinese herbal name, Huangqi), dried roots of Astragalus membranaceus (Fisch.) Bge.var. mongholicus (Bge.) Hsiao; (2) the carda part of Radix Angelicae Sinensis root (Guiwei), the dried lateral roots of Angelica sinensis (Oliv.) Diels; (3) Radix Paeoniae Rubra (Chishao), the dried roots of Paeonia lactiflora Pall.; (4) Rhizoma Chuanxiong (Chuanxiong), the dried rhizomes of Ligusticum chuanxiong Hort; (5) Flos Carthami (Honghua), the dried flowers of Carthamus tinctorius L.; (6) Semen Persicae (Taoren), the dried seeds of Amygdalus persica L.; and (7) Pheretima (Dilong), and the dried bodies of Pheretima aspergillum (E. Perrier), in the ratio of 120:6:4.5:3:3:3:3 on a dry weight basis. All of these components are recorded in the Chinese Pharmacopoeia (2005 Edition) [1]. This medicine has been widely used in Chinese clinical practice for treatment and prevention of ischemic cardio-cerebral vascular diseases and stroke-induced disability for thousands of years. According to the traditional Chinese medical literature, it is used to enhance blood circulation and activate energy (qi) flow through energy meridians.

Recent studies show that BYHWT also provides neuroprotective effects for conditions such as brain ischemia, stroke-induced disability [2] and act against cerebral ischaemia-reperfusion (CI/R) injury [3,4]. This formula has also been shown to have the potential improvement for stroke and extended lifespan, primarily by regulating inflammation, apoptosis, angiogenesis and blood coagulation; and up-regulation by mediating neurogenesis and nervous system development [5]. The previous clinical evidence indicated that the ameliorative effects of BYHWT on coronary heart disease with qi deficiency and blood stasis syndrome in rats was related to the inhibition of C-reactive protein and CD40 gene and the regulation of endothelium-derived vasoactive factors [6]; and it was regulating the improvement of hemorheological disorders and energy metabolism [7].

**Figure 1:**
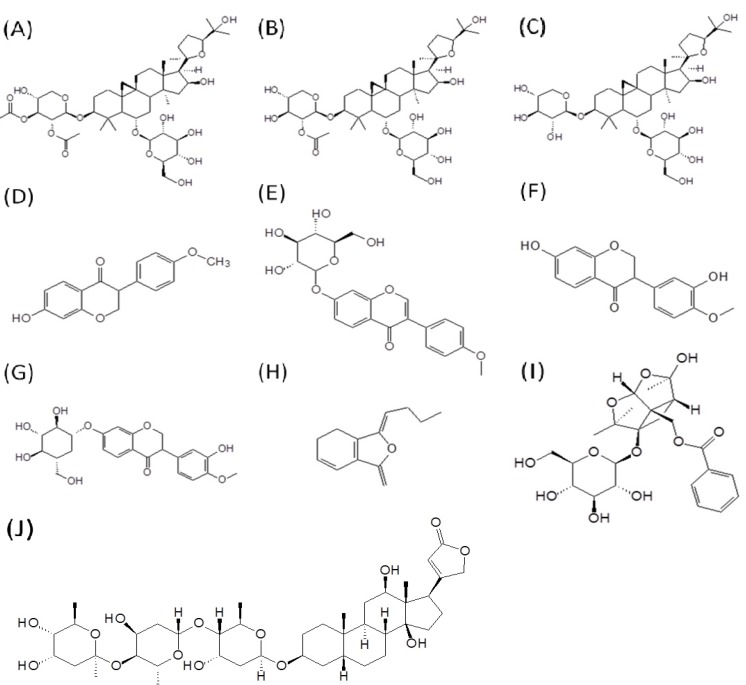
The structures of nine active compounds in bu-yang-huan-wu-tang: (**A**) astragaloside I; (**B**) astragaloside II; (**C**) astragaloside IV; (**D**) formononetin; (**E**) ononin; (**F**) calycosin; (**G**) calycosin-7-*O*-β-D-glucoside; (**H**) ligustilide and (**I**) paeoniflorin (**J**) digoxin.

Although many beneficial effects have been found, the actual bioactive ingredients of BYHWT need to be clarified. Several methods have been previously described to determine the major effective components related to pharmacological functions [8,9], including hepatoprotective effects, neuroprotective effects against ischemic brain injury, hematopoietic, antioxidative, antihypertensive, immunological properties, cardiotonic, and anti-aging activities [10]. Among its components, alkaloids and glycosides are the most representative of BYHWT in terms of both the proportion of the contents and their biological activities. Radix astragali contains mainly components such as saponins, isoﬂavones, polysaccharides and amino acids. The saponin components include astragaloside I, II, III, IV, V, VI, VII, VIII, *etc*. [11]. Astragaloside IV has been used as the marker compound of Radix Astragali for quality identification; and it inhibited vessel contraction through blocking calcium inﬂux and intracellular calcium release, actions that were attributed mainly to the endothelium-dependent NO-cGMP pathway [12]. The amounts of major isoflavonoids in Radix Astragali were formononetin, ononin, calycosin and its glycoside [13], which boost energy, strengthen the immune system, promote health activities and promote skin growth [14]. Both saponins and flavonoids should be considered as marker compounds for the chemical evaluation or standardization of Radix Astragali preparations [15]. Ligusticum chuanxiong mainly contained tetramethylpyrazine, perlolyrine, ligustilide, ferulic acid, protocatechuic acid, *etc*. [11]. Ligustilide, a phthalide derivative, is the most abundant constituent in the herb and is the most abundant bioactive ingredient in Rhizoma Chuanxiong and Angelica sinensis for quality identiﬁcation purposes. In addition, the vasodilatation, antiplatelet aggregation, antithrombotic, serotonergic activity, and antiproliferative properties of ligustilide have been well documented [16,17]. Paeonia lactiflora is mainly comprised of the effective glycoside composition of paeoniﬂorin, oxypaeoniﬂorin, benzoylpaeoniﬂorin, *etc*. [11]. Paeoniﬂorin is the marker compound in Radix Paeoniae Rubra [18,19]. Previous experimental studies have suggested a close relationship between these components and the bioactive mechanism of BYHWT. The chemical structures of the nine major compounds are shown in Figure 1.

Due to the co-existence of multiple bioactive components in traditional Chinese medicinal products, it is not sufficient to monitor only one component for the quality control of its raw materials and proprietary traditional Chinese medicine products. Compared with conventional analytical approaches, high-performance liquid chromatography (HPLC) coupled with tandem mass technique emphasize the integral characterization of a complex system with a degree of quantitative reliability. Using this method, a particular herbal preparation with complex constituents can be identified and distinguished. As a novel approach to identify and control the quality of Chinese medicines, chromatographic techniques are regarded as some of the most powerful and rational analytical instruments for the quality evaluation of herbal preparations. The technology has been widely accepted and has attracted ever-increasing attention due to both the high detection sensitivity and high separation efficiency of the technique [20]. Thus, the recognition methods such as multi-ingredients quantitative analysis should be taken into consideration for reasonable definition of herbal medicine. Since the chemical constituents of the formula in BYHWT are complex, this study focused on the main active constituents. In particular, astragaloside I, astragaloside II, astragaloside IV, formononetin, ononin, calycosin, calycosin-7-*O*-β-d-glucoside, ligustilide and paeoniflorin were used in the quality control studies [21]. These investigations were performed for a better understanding of this compound’s commercial material and by comparing the six commercial BYHWT products for the quantification of the marker chemical compositions in each one. The ESI-MS/MS fragmentation generates the product ion and daughter ion, which lead to the development of an MRM method with highest sensitivity and selectivity.

The present study is to discuss new improvements in strategies and methodologies for BYHWT’s multi-component evaluation. The contents of nine components in BYHWT from different manufacturers were determined to establish the effectiveness of the method. Different compositions may have significant differences in their safety and efficacy for different health problems. By making a thorough investigation of this compound’s chemical compositions, its concentrations of crucial constituents, stability, structure-activity relationships and mechanisms behind the beneficial and toxic effects has been extended to support future investigation.

## 2. Results and Discussion

### 2.1. Optimization of Chromatographic Separation Conditions

The great structural diversity of active compounds in the BYHWT formula makes it difficult to obtain good separation performance for all chemical compounds during analysis. Due to the complex constituents in the herbal medicine, the analysis of such a complex mixture is a great challenge for pharmaceutical analysts. The tandem mass spectrometric detection, however, has led to a remarkable improvement in detectability and selectivity by engaging MRM acquisition [21,22]. Due to the high selectivity of MRM mode, accurate methods and optimization of chromatographic separation are greatly simplified. Furthermore, precursor and product ion monitoring can be used to increase the specificity of detection and identification of target constituents in the samples. In the current study, each standard solution of analyte was infused into the chromatographic system, and the ESI source parameters were investigated using positive ion modes for simultaneous quantification of herbal compounds. Glycosides could be analyzed in both positive and negative ionization. However, most signals of phthalides, lactones and triterpenes were clear in the positive ion mode, and so both positive ion modes had to be employed to confirm the corresponding selection. The results show that ESI in the positive ion mode is better and has excellent signal sensitivity. The maximum signal intensity was obtained by adjusting the energy of declustering potential, focusing potential, entrance potential, collision energy and collision cell exit potential. The HPLC-MS/MS with ESI positive ionization and the MRM mode provided a highly selective method for the determination of nine target compounds and the internal standard.

**Figure 2:**
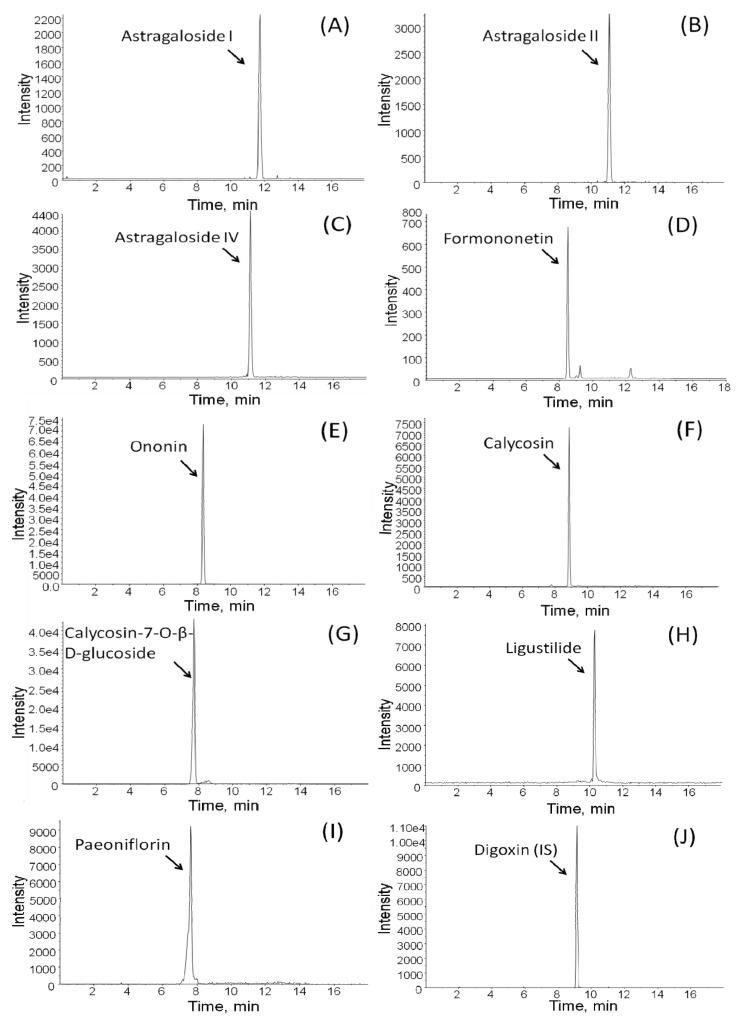
The typical MRM chromatograms of nine standard solution (**A**) astragaloside I; (**B**) astragaloside II; (**C**) astragaloside IV; (**D**) formononetin; (**E**) ononin; (**F**) calycosin; (**G**) calycosin-7-*O*-β-d-glucoside; (**H**) ligustilide and (**I**) paeoniflorin and (**J**) digoxin (IS).

To confirm the structure of the investigated compounds, the mass spectral data of these compounds were recorded and the precursor ion and major fragment ions were examined. The precursor and product ions of nine reference compounds were selected by infusing individual standard solutions into the mass spectrometer. All analytical condition of LC-MS/MS for the identification of astragaloside I, astragaloside II, astragaloside IV, formononetin, ononin, calycosin, calycosin-7-*O*-β-D-glucoside, ligustilide, paeoniflorin and internal standard are shown in Table 1. Digoxin was the internal standard used in the quantitative method. Column separations, mobile phase compositions, gradient elution process, flow rate of the mobile phase, and temperature were performed to achieve an accurate analysis for as many analytes as possible on the Phenomenex^®^ Gemini C18 column. The chromatogram of the standard compounds is shown in Figure 2, for which a standard mixture solution of astragaloside I, astragaloside II, astragaloside IV, formononetin, ononin, calycosin, calycosin-7-*O*-β-d-glucoside, ligustilide, paeoniflorin and digoxin was used. Under the optimized linear gradient mode, the number of peaks on the chromatogram was achieved within 18 min. The HPLC-ESI-MS/MS analysis was performed on both standards and samples, which could be utilized for the identification of the target compounds. Nine investigated analytes in the extracts of BYHWT were comprehensively compared by the retention time and ion fragments with those of authentic standards.

In the precursor ion scan mass spectra, most analytes formed molecular ions [M + NH_4_]^+^, except that the ions of formononetin, ononin, calycosin and calycosin-7-*O*-β-D-glucoside were [M + H]^+^. Astragalosides represent the major compounds responsible for the bioactivities of Radix astragali, and so the qualitative and quantitative analysis of astragalosides should be observed. In the present study, the three main astragalosides of astragaloside I, astragaloside II and astragaloside IV were selected for analysis. It has been reported that these peaks require careful identification and separation, because all of them belong to the cycloartane-type triterpene glycosides [23]. The analysis of astragaloside I achieved a positive ion of *m/z* 886.6, referring to the [M + NH_4_]^+^ precursor ion. Consequent product ion scan of precursor ion produced a fragmentation pattern dominated by an ion at *m/z* 143.2. Thus, the transition *m/z* 886.6→143.2 was selected for the identification and quantification of astragaloside I. Due to their identical isomer cycloartane-type triterpene structures, astragaloside II and astragaloside IV had similar fragmentation patterns. Similarly, the MRM transitions *m/z* 844.5→143.2 and *m/z* 802.6→143.2 were selected to monitor astragaloside II and astragaloside IV, respectively.

**Table 1:** The analytical condition of LC-MS/MS for the identification of the nine compounds and internal standard.

Compounds	RT (min)	Mass fragments		Ionization parameters				
		Precursor (amu)	Product (amu)	DP (V)	FP (V)	EP (V)	CE (V)	CXP (V)
Astragaloside I	11.21	886.6 [M + NH_4_]^+^	143.2	55	290	12	23	13
Astragaloside II	10.73	844.5 [M + NH_4_]^+^	143.2	62	330	13	25	13
Astragaloside IV	11.18	802.6 [M + NH_4_]^+^	143.2	65	370	14	28	10
Formononetin	8.32	269.2 [M + H]^+^	253.2	190	380	14	37	16
Ononin	8.23	431.3 [M + H]^+^	269.2	80	380	13	20	18
Calycosin	8.72	285.2 [M + H]^+^	270.2	190	380	13	33	17
Calycosin-7-O-β- d-glucoside	7.68	447.2 [M + H]^+^	285.1	80	380	12	23	19
Ligustilide	10.05	208.1 [M + NH_4_]^+^	173.2	70	380	14	25	11
Paeoniflorin	7.37	498.3 [M + NH_4_]^+^	179.2	76	390	14	25	12
Digoxin (IS)	9.12	798.6 [M + NH_4_]^+^	651.6	20	160	10	18	11

The major compounds of formononetin, ononin, calycosin and its glycoside were found to be iso-flavonoids, and their fragment ions were consistent with their structure and arose from cleavage of the related glycoside or substituent group. The chemical structure of formononetin resembled that of calycosin, differing in the substituent group located on a ring with a hydroxyl group [24]. The calycosin fragmentation could be related to other isoflavones. The protonated isoflavone aglycones resulting from loss of a methyl radical were the predominant fragmentations for the aglycones, causing the formation of very stable cation radical structures. The precursor molecular ion [M + H]^+^ of formononetin and the main fragment resulting from loss of a hydroxyl group are expected to be found at *m/z* 253.2. The compounds ononin and calycosin-7-*O*-β-D-glucoside both are *O*-glycosides. In positive ion mode, the glycosidic bonds of *O*-glycosides were easily cleaved to produce daughter ions of [M + H − 162]^+^ at *m/z* 269.2 and 285.1 by the facile neutral loss of a glycoside residue from the corresponding protonated molecule ions at *m/z* 431.3 and 447.2, respectively [25].

The major product ions of ligustilide in Radix Angelicae Sinensis and Rhizoma Ligustici Chuanxiong at *m/z* 173.2 corresponded to one hydroxyl group loss from the precursor ion. The major product ion of *m/z* 179.2 for paeoniflorin in Radix Paeoniae Rubra may be due to the loss of a glucose molecule, a benzoic acid molecule and a hydrogen molecule [26]. A compound with similar physical-chemical properties should be considered as the internal standard for analysis. Digoxin was chosen as the internal standard in the quantitative method, because it has a similar chemical structure as astragaloside I, astragaloside II and astragaloside IV. Digoxin is applicable to the investigated analytes in its chromatographic recovery and ionization properties.

The different types of column used for investigation were: Agilent Zorbax^®^-SB-C3 (150 mm × 4.6 mm I.D, 5 µm), Kromasil C18 (5 μm, 250 mm × 4.6 mm), and Phenomenex^®^ Gemini C18 (5 µm, 150 mm × 2.6 mm). All three types of column exhibited different chromatography behavior. The Phenomenex^®^ Gemini C18 column was selected for use because it exhibited better separation than the other columns. The advantage of the gradient elution system was optimized in detailed separations, and it related the nine selected marker compounds with the internal standard over a broad range of polarity. The gradient elution had a significant influence on the separation of the multiple compounds. The chromatographic conditions achieved good resolution and appropriate ionization in the presence of endogenous species and co-elution. Experimental trials used several mobile phase compositions, including mixtures of methanol/water or acetonitrile/water and different additives such as ammonium acetate or formic acid at different concentrations. An acidified mobile phase could minimize peak tailing, improve resolution, and facilitate ionization. It was observed that addition of 0.1% formic acid in the mobile phase provided a higher response and better intensity. The chemical structures of astragalosides and internal standard have been reported as being relatively stable to acids [27]. The acetonitrile-water system provides good separation for the investigated compounds, but the peak of the compound ligustilide seriously interfered with the performance in our tests. Thus a gradient of methanol-NH4OAc with 0.1% formic acid was selected as the most appropriate eluent with a flow rate of 0.2 mL/min.

### 2.2. Validation of the HPLC Procedure

The results of regression equations and linear range for the calibration curves are shown in Table 2. The calibration curves were calculated by the peak-area ratio of each analyte to the internal standard *versus* the concentration of each analyte. To avoid bias to the low concentrations of the standard curve caused by the high concentrations, the calibration curves were separated in different ranges. Within the linear range, the calibration curve had good linearity (r^2^ > 0.995) for each analyte. The intra-day and inter-day precision and accuracy of the assay method are shown in Table 3. Both intra-day and inter-day precisions for all the nine compounds were below 11.6%. The accuracy at three different concentrations was within the range of −9.6% to 11.5%. The standard stability was examined. Standard samples of each analyte were analyzed respectively at low, middle and high concentrations. Variations of concentrations of nine compounds were calculated, and the stability values are shown in Table 4, indicating a reasonable stability of the standard solutions. The stability profile of the BYHWT extract and commercial pharmaceutical herbal products in 6 h was also determined. The results show that there were no significant changes to these compounds during the 6 h room temperature period. In addition, the reproducibility results shown in Table 5 show that the range of RSD for the nine compounds was from 0.1% to 12.9%, indicating that the consistency of the level extracted from an individual source was within 15%. The above results demonstrate the reliability of the measurement of these samples. In summary, a rapid and simple HPLC-MS/MS procedure was developed and validated for estimation of BYHWT extract and commercial samples.

**Table 2:** Regression equation, correlation coefficient, linear range, limits of quantification and limit of determination of nine compounds.

Compounds	Regression equation	Correlation coefficient	Linear range (ng/mL)	Low limits of quantification (ng/mL)	Limits of determination (ng/mL)
Astragaloside I	y = 558.86x − 734.12	0.997	0.5–500	0.5	0.17
Astragaloside II	y = 916.57x + 4154.8	0.999	0.5–500	0.5	0.17
Astragaloside IV	y = 1050.1x + 5612.8	0.998	0.5–500	0.5	0.17
Formononetin	y = 219.53x − 1076.2	0.997	5–1000	5	1.65
Ononin	y = 24939x + 233299	0.999	5–1000	5	1.65
Calycosin	y = 2917.2x − 5405	0.998	1–500	1	0.33
Calycosin-7-O-β- d-glucoside	y = 10233x − 12150	0.998	10–1000	10	3.3
Ligustilide	y = 7634.8x + 17949	0.997	5–500	5	1.65
Paeoniflorin	y = 6093.8x − 30867	0.998	10–1000	10	3.3

**Table 3:** Intra-day and inter-day precision and accuracy for the determination for nine compounds from standard samples of nine active compounds (*n* = 6).

Nominal concentration (ng/mL)	Intra-day			Inter-day		
	Observed concentration (ng/mL)	Precision (%)	Accuracy (%)	Observed concentration (ng/mL)	Precision (%)	Accuracy (%)
Astragaloside I						
10	11.3 ± 0.1	0.6	5.2	9.4 ± 0.32	3.1	1.4
100	105 ± 2.7	4.3	3.9	101 ± 6.9	4.2	−5.3
1000	1007 ± 6.8	7.4	−7.8	1032 ± 9.1	11.6	8.8
Astragaloside II						
10	12.3 ± 0.3	4.2	4.2	0.5 ± 1.8	1.5	2.3
100	99.8 ± 3.4	5.5	5.8	101 ± 3.1	5.2	7.2
1000	1002 ± 11.2	9.0	11.5	101 ± 5.4	6.8	5.9
Astragaloside IV						
10	11.1 ± 0.8	4.8	1.8	9.8 ± 0.7	4.4	3.2
100	102 ± 2.3	4.2	3.2	103 ± 4.3	3.6	5.4
1000	1008 ± 6.4	7.2	4.8	1011 ± 5.2	4.8	6.6
Formononetin						
10	11.3 ± 2.3	6.2	3.4	9.78 ± 5.2	3.6	2.8
100	104 ± 5.8	5.8	5.6	96.7 ± 5.5	3.5	−1.2
1000	992 ± 12.2	3.8	2.6	1004 ± 18.2	7.2	3.4
Ononin						
10	9.5 ± 4.4	4.2	3.4	9.3 ± 1.7	5.8	1.8
100	106 ± 4.5	3.6	−6.2	−96.5 ± 7.1	4.3	−2.4
1000	1009 ± 5.3	3.8	7.4	1003 ± 12.7	3.4	1.6
Calycosin						
10	12.2 ± 8.1	7.68	3.2	9.45 ± 1.3	6.4	5.6
100	104 ± 6.3	4.92	1.2	101 ± 3.5	2.5	2.2
1000	1011 ± 3.1	5.76	1.8	1016 ± 9.4	4.2	4.8
Calycosin-7-O-β-d-glucoside						
10	11.5 ± 0.3	2.1	1.8	9.86 ± 1.4	5.3	2.3
100	105 ± 5.4	4.3	−1.3	107 ± 7.6	4.29	−7.9
1000	1009 ± 6.9	7.6	3.6	1011 ± 13.3	8.5	5.3
Ligustilide						
10	9.5 ± 1.6	6.9	0.3	10.3 ± 2.2	4.9	2.7
100	103 ± 2.1	5.8	2.4	9.43 ± 5.7	9.2	6.3
1000	1007 ± 5.5	3.6	11.4	1008 ± 8.6	5.34	10.3
Paeoniflorin						
10	10.7 ± 0.45	1.5	−1.4	11.5 ± 0.3	0.9	1.8
100	98.3 ± 8.29	4.8	3.8	104 ± 4.2	8.6	−0.3
1000	995 ± 5.78	5.7	8.3	1005 ± 11	4.8	−9.6

**Table 4:** Stability of the nine compounds of herbal medicine.

Concentration (μg/mL)	Stability (%)		
	Standard solution	BYHWT Extraction	Commercial product A
Astragaloside I			
1	7.7 ± 2.4	4.9 ± 3.4	5.2 ± 2.1
10	4.5 ± 6.3		
100	3.9 ± 5.0		
Astragaloside II			
1	12 ± 5.3	8.8 ± 5.6	5.2 ± 4.4
10	6.3 ± 8.8		
100	3.4 ± 7.8		
Astragaloside IV			
1	9.4 ± 7.4	3.4 ± 6.2	10.1 ± 3.5
10	5.3 ± 4.6		
100	4.4 ± 7.2		
Formononetin			
10	10 ± 4.1	5.8 ± 3.1	6.2 ± 5.1
100	6.6 ± 2.1		
1000	2.8 ± 12		
Ononin			
1	6.2 ± 3.4	7.2 ± 4.7	11.2 ± 5.1
10	3.0 ± 2.5		
100	3.1 ± 2.8		
Calycosin			
10	4.5 ± 6.3	5.8 ± 3.8	6.3 ± 4.1
100	3.5 ± 2.7		
1000	4.1 ± 5.3		
Calycosin-7-O-β-d-glucoside			
1	8.5 ± 10	12.6 ± 3.5	6.8 ± 5.1
10	5.2 ± 6.6		
100	4.5 ± 3.5		
Ligustilide			
1	7.0 ± 4.3	4.6 ± 3.7	7.5 ± 4.8
10	4.8 ± 5.6		
100	3.2 ± 5.8		
Paeoniflorin			
10	6.3 ± 7.8	9.3 ± 5.2	5.7 ± 2.7
100	4.6 ± 3.4		
1000	2.7 ± 4.9		

Data are expressed as means ± S.D. (*n* = 3).

**Table 5:** Reproducibility of the nine compounds in BYHWT extract and A–F stands for the different commercial pharmaceutical herbal products.

mg/g ( *n* = 3)	BYHWT Extraction	A	B	C	D	E	F
Astragaloside I	0.8	0.8	0.1	2.8	0.2	4.5	0.6
Astragaloside II	1.2	2.9	2.4	5.0	1.7	8.4	6.4
Astragaloside IV	4.4	12.9	1.1	11.4	2.4	7.3	5.0
Formononetin	0.0	1.9	0.7	3.5	2.6	7.3	2.8
Ononin	1.6	7.9	7.5	10.0	2.9	3.6	3.3
Calycosin	0.1	0.8	0.5	4.9	0.2	3.0	1.8
Calycosin-7-O-β-D-glucoside	0.4	7.1	1.1	3.9	3.4	1.4	4.4
Ligustilide	3.0	4.0	0.7	2.5	3.1	4.0	5.0
Paeoniflorin	1.7	0.2	0.1	2.3	4.4	0.1	6.4

Data expressed as mean (*n* = 3).

### 2.3. Application to Analysis of BYHWT Commercial Samples

The contents of nine major compounds in commercial BYHWT samples could be readily determined fairly accurately by the method described above. The results obtained from the six brands of commercial BYHWT pharmaceutical herbal products are presented in Table 5. Even though the six brands of commercial pharmaceutical herbal products had the same ingredient composition of crude drugs, most products were prepared with different manufacture methods from crude drugs. Thus the contents of active compounds varied among them. Extraction powder is the original dosage form of BYHWT. Triterpene glycosides of astragaloside I, II, IV; the isoflavonoids of formononetin, ononin calycosin, calycosin-7-*O*-β-d-glucoside; ligustilide and paeoniflorin were also found in the six products of the BYHWT commercial pharmaceutical herbal products, indicating that the manufacturing processes could retain the major constituents of the seven crude drugs, and the main components of this preparation could be well preserved during the extraction.

In these commercial pharmaceutical herbal products, the amounts of constituents, especially the most abundant and effective nine active compounds were much lower than seen with our BYHWT extraction, probably as a result of the difference in raw materials used and the starch granulation process. Comparing the sample amount of the nine active compounds between the BYHWT commercial products, product B had the highest level of the nine active compounds and Product F had the lowest. These commercial formulae were obviously manufactured such that there were different herb extraction yields to the decoctions.

Comparing the sample amount of triterpene glycosides, Table 5 shows that the contents of astragaloside I differed significantly, with the highest levels being 2.46, or 0.78 mg/g in BYHWT extract and sample B, respectively, and the content of astragalosie IV with the low level of 0.54 and 0.19 mg/g in BYHWT extract and sample B. In all tested samples, astragaloside I and astragaloside II contents were greater than or equal to that of astragaloside IV, for both the The BYHWT extraction and the commercial products. Astragaloside IV is the major active constituent of Astragalus membranaceus. Radix Astragali of astragaloside I, astragaloside II and astragaloside IV are comprised of the same aglycone cycloastragenol and differ only in the number and position of glucosyls, but their content were significantly different in our results. According to a previous report, the astragaloside profiles of Radix Astragali differed according to the regions in which the herb was grown, primarily Dongying, Hunyuan and Tongyuan. It can be observed that astragalosides I, II and IV were the main astragalosides in Radix Astragali but that their proportions are clearly different in material according to the regions in which they were grown. The contents of astragaloside I: astragaloside II: astragaloside IV were in the approximate proportions of high:low:medium from Dongying, high:low:low from Hunyuan, and medium:low:high from Tongyuan. This indicates that the different profiles of astragalosides were related to the material origins. Astragalosides are the primary indicators that can be employed for quality control purposes [28]. The most important is the proportion of astragaloside IV to astragaloside I, which could be selected as a characteristic parameter for the fingerprints of A. mongholicus and A. membranceus. Due to the complexity of TCMs, co-existing compounds with similar structural isomers are also common. It was noticed that the structural isomers showed significant sample-to-sample variability in the amounts of isoflavonoids measured. In our results, the contents of each studied isoflovonoid showed marked variations between the different products. Both formononetin and calycosin contain same backbone structure, and our results indicated high levels of formononetin and low levels of ononin in all products. Both ononin and calycosin-7-*O*-β-d-glycoside are *O*-glycosides; and our results showed that the amount of calycosin-7-*O*-β-d-glucoside was highest in the reference samples, while the ononin levels were lowest among the isoflovonoids. This might be due to the raw material used or the procedure of extraction and pharmaceutical preparation. It has been observed that the level of each isoflovonoid from different commercial pharmaceutical herbal products showed a similar extent of variation. Ligustilide was the most dominant component in Chuanxiong and Angelicae. Pharmaceutical product F contained the least ligustilide and others also had low levels of BYHWT. The paeoniflorin in Paeoniae contributes to the high content among the samples tested. Furthermore, despite the variation of paeoniflorin, the levels in all of the products were high.

Examining the chemical composition of these samples provides useful information for the evaluation of BYHWT. These results suggest that there are significant differences in the chemical composition and possible differences in medicinal effects between commercial pharmaceutical products, indicating the need for quality control of commercial botanically based herbal medicine ingredients.

## 3. Experimental

### 3.1. Materials and Reagent

Six brands, labeled A–F, of commercially available ready-made TCM preparations of BYHWT were collected in Taiwan from different well-known pharmaceutical companies, and all compounds were of high quality according to the GMP for Chinese herbal medicines. The crude drugs of six brands were comprised of Radix Astragali, the carda part of Radix Angelicae Sinensis root, Radix Paeoniae Rubra, Rhizoma Chuanxiong, Flos Carthami, and the dried bodies of Pheretima aspergillum in the ratio of 120:6:4.5:3:3:3:3. The mixing crude drugs were elaborated by extracting, concentrating, drying, and with finely granulation processes in different composition of crude drug (g), extract (g) and starch granulation (g) as: A (16: 4: 3), B (9.6: 2: 1), C (4.29: 1: 1), D (4.84: 1.1: 1), E (5.3: 1: 1), F (12: 2: 1). The manufacture is allying modern scientific technology to the classical way of making scientifically processed Chinese herbal medication (powdered). The reference standards of astragaloside I, astragaloside II, formononetin, calycosin, and calycosin-7-*O*-β-D-glucoside were purchased from the Shanghai Tauto Biotech Co. LTD. (Shanghai, China) and stored at 4 °C. Ononin was supplied from Extrasynthese (Genay Cedex, France). Ligustilide was obtained from ChromaDex. Inc., CA 92618, USA (10 mg/mL in acetonitrile). Paeoniflorin was from Nacalai Tesque, Inc. (Kyoto, Japan). Astragaloside IV, digoxin (internal standard) was provided by Sigma-Aldrich Chemicals (St. Louis, MO, USA). Ethanol was purchased from Taiwan Tobacco & Liquor Corporation (Taipei, Taiwan). Ammonium acetate, formic acid, acetonitrile, and methanol were of HPLC grade for analysis grade from E. Merck (Darmstadt, Germany). Triply deionized water prepared by Millipore (Bedford, MA, USA) was prepared for all aqueous solutions.

### 3.2. Preparation of Bu-yang-hwan-wu-tang Extract

The crude drugs contained the following components: dried roots of Astragalus membranaceus, dried lateral roots of Angelica sinensis, dried roots of Paeonia lactiflora Pall, dried rhizomes of Ligusticum chuanxiong Hort, dried flowers of Carthamus tinctorius L., dried seeds of Amygdalus persica L. and dried bodies of Pheretima aspergillum. All were purchased from an herbal drugstore in Taipei, Taiwan and verified by the lab of Professor Lie-Chwen Lin at the National Research Institute of Chinese Medicine, Taipei, Taiwan. The weights of Radix Astragali (1,800 g), Radix Angelicae sinensis (90 g), Radix Paeoniae rubra (90 g), Rhizoma Chuanxiong (45 g), Semen Persicae (45 g), Flos Carthami (45 g), and Phizoma Chuanziong (45 g) were pounded into small pieces. The herbal admixture was extracted with 50% ethanol in a water bath (70 °C, 6 L) for 3 h by a reflux apparatus. The residue was re-extracted with fresh 6 L 50% ethanol and the procedure was repeated. The combined ethanolic extract was concentrated using a rotary vacuum evaporator and water was removed by freeze drying. This process resulted in 366 g of crude extract of BYHWT. The BYHWT portion was dissolved in methanol and filtered through a 0.22 μm syringe filter prior to HPLC-MS/MS analysis.

### 3.3. Liquid Chromatography-Tandem Mass Spectrometry

An Agilent-1100 HPLC system (Agilent Technologies, Waldbronn, Germany) with an Applied Biosystems/MDS Sciex API 3000 tandem quadrupole mass spectrometer (Framingham, MA, USA) equipped with quaternary pump, injector, vacuum degasser, and autosampler was used. The chromatographic separation was performed on a Phenomenex^®^ Gemini C18 (150 mm × 2.0 mm I.D, 5 μm particles) column with the column temperature set at 25 °C. The mobile phase consisted of ammonium acetate buffer (0.1% formic acid, A) and methanol 10 mM (0.1% formic acid, B) with a linear gradient: 0–1 min: 30%–70% A; 1–2 min 70%–90%; 2–8 min: 90%–90%; 8–9 min: 90%–30%, 9–18 min: 30%–30%, v/v. The flow rate was 0.2 mL/min, and the injection volume was 10 μL. A 0.2 mL/min portion of the column effluent was delivered into the ion source of the mass spectrometer. The Applied Biosystems/MDS Sciex API 3000 tandem quadrupole mass spectrometer equipped with electrospray ionization (ESI) turbo ion interface was used with the following parameters: nebulizer gas:10, curtain gas: 7, collisionally activated dissociation gas: 12, ionspray voltage: 5,000 V, turbo ionspray temperature: 450 °C. Nitrogen was used in all cases. The mass spectrometer was operated in the positive ion detection mode. Analytes were quantified by multiple-reaction monitoring (MRM) mode performing with the precursor-to-product ion pair.

### 3.4. Preparation of Calibration Standards

Each accurately weighed authentic standards (astragaloside I, astragaloside II, formononetin, ononin, calycosin, calycosin-7-O-β-d-glucoside and paeoniflorin) were dissolved in methanol for a final concentration of 1 mg/mL, and then a mixed methanolic stock solution of standards was prepared. The authentic standard of astragalosdie IV was dissolved in 50% (v/v) acetonitrile to make a 1 mg/mL stock solution, and ligustilide was prepared in 100% acetonitrile of 10 mg/mL stock solution. The internal standard stock solution of 1 mg/mL was also prepared in 50% (v/v) acetonitrile. A set of standard solutions were prepared by appropriate dilution of the stock solution with methanol, in order to make the calibration curve. All the solutions were stored at −20 °C in a freezer. The samples were prepared to final concentrations at the selected ranges. The calibration standard curves were obtained by least-square linear regression of the peak area *versus* the concentrations. All calibration curves were required to have a correlation value of at least 0.99. The concentration of analytes were derived from the correlated calibration curve and corrected by respective dilution volume. For a standard curve, the ratios of the chromatographic peaks area (standard analytes/internal standard) as ordinate variables were plotted *versus* the concentration. The validation samples were also prepared in the same way (10, 100, 1,000 ng/mL) at low, middle and high concentrations.

### 3.5. Method Validation

The specificity evaluation showed the presence of interfering peaks at the retention time of the analytes of the response. The response for the interfering peaks at the retention time of the internal standards was also considered as part of the response in the concentration. The precision and accuracy of the method was determined for intra- and inter-day validations. The intra-day variability was performed in six replicates on the same sample extracted during a single day, while the inter-day was carried out in other independent samples extracted on six consecutive days. The ratios of observed concentration and nominal concentration of the mixed standard solutions were calculated to determine accuracy. The analogs were added at six concentration levels (10, 100, 1000 ng/mL) at low, middle and high concentrations. The inter-day and intra-day accuracy and precision value for the lowest permissible reproducibility concentrations were defined as being within ±15%. To determine the limits of detection and quantification, methanol stock solutions containing the reference compounds were diluted to a series of appropriate concentrations with the same solvent. The limits of detection (LOD) and quantification (LOQ) under the present chromatographic conditions were determined at S/N ratios (the ratio of signal to noise) of 3 and 10, respectively.

According to the guidance for industry from the FDA, stability procedures should evaluate the stability of the analytes during sample collection and handling. In our experiment, the period from preparation of calibration standards till injecting to LC/MS was with only a few hours. Hence, sample stability during transport and handling was considered under laboratory conditions within 6 h. Standard solution stability was determined by placing the samples at room temperature for 6 h, and assessed by using the low, middle and high concentrations of analytes. Stability of BYHWT extract and a commercial sample were evaluated by analyzing samples kept at room temperature for 6 h (C_t__=__6_). All stabilities were calculating as the ratio of average concentration and freshly prepared samples (C_t__=__0_), and the calculation was as follow: Stability (%) = [(C_t__=__0_ − C_t__=__6_)/C_t__=__0_] × 100. In addition, in order to test the reproducibility of extraction, commercial samples of BYHWT were extracted independently in triplicate to evaluate the reproducibility of the extraction protocol.

### 3.6. Sample Preparation of BYHWT Commercial Products for Quantitation

The BYHWT powdered samples of our lab-prepared extract and commercial pharmaceutical herbal products (A–F) were accurately weighed and extracted using 50 mL of 100% methanol for 60 min at 4 °C with sonication. The slurry mixture was centrifuged at 6,000 rpm for 10 min. The supernatant was filtered through a 0.22 μm syringe filter and made a final concentration of 20 mg/mL (equivalent to dry weight of raw materials). The samples were kept frozen at 4 °C for HPLC-MS/MS analysis. Quantitation of composition was directed in triplicate with data reported as mean ± standard deviation.

## 4. Conclusions

In this study, a total of nine active compounds in the Chinese medicine formula BYHWT extract and commercial samples of this medicine were successfully determined by liquid chromatography with tandem mass spectrometry detection. Based on the analytical method, HPLC-MS/MS with MRM modes represents a powerful technique to analyze the multiple ingredients of medical formulae. The results indicate that commercial BYHWT products differ significantly differ between brands in their bioactive components and activities, indicating the need for a quality assurance mechanism for commercial BYHWT and its derived active products.
